# A retained foreign body in a jejunal diverticulum

**DOI:** 10.1093/jscr/rjad217

**Published:** 2023-04-28

**Authors:** Joseph Phillipos, Joseph Jaya, Chris Mills

**Affiliations:** Department of General Surgery, La Trobe Regional Hospital, Traralgon 3844, Australia; Department of General Surgery, La Trobe Regional Hospital, Traralgon 3844, Australia; Department of General Surgery, La Trobe Regional Hospital, Traralgon 3844, Australia

**Keywords:** Jejunal diverticulum, hearing aid, lithium battery, retained foreign body

## Abstract

Retained foreign bodies in small bowel diverticula are a rare occurrence. Batteries and battery containing products can cause bowel perforation, and when retained in diverticula, retrieval methods may need to be considered. We present a novel case of a hearing aid powered by lithium battery getting stuck in a jejunal diverticulum, and outline an approach to retrieval.

## INTRODUCTION

Foreign bodies can become lodged in diverticulum, and can result in tissue necrosis and perforation. Foreign body retention is rare in jejunal diverticula compared to Meckel’s diverticula, possibly due to the wider neck of jejunal diverticula, allowing easier outflow [1]. Nevertheless, foreign bodies in jejunal diverticula may need to be retrieved if they are slow to pass naturally, especially when they contain corrosive substances like lithium batteries.

## CASE REPORT

**Figure 1:**
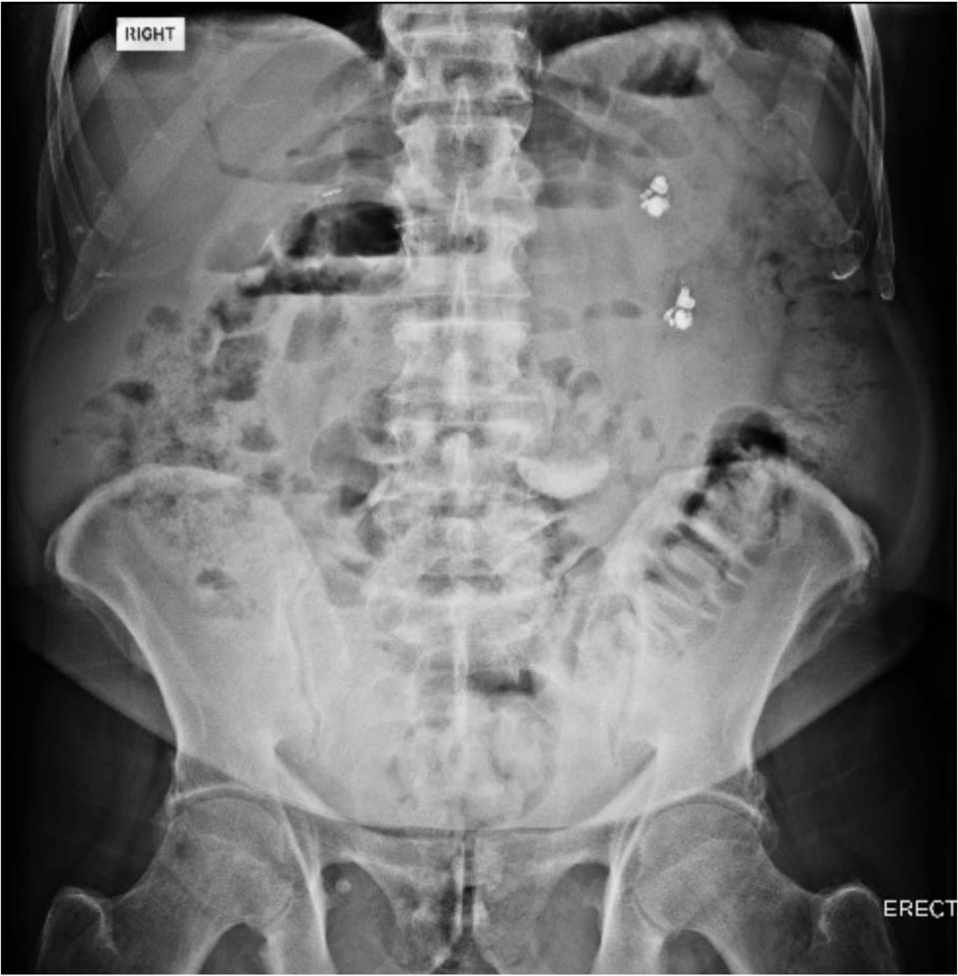
Abdominal X-ray identifying two hearing aids on day of initial presentation.

**Figure 2:**
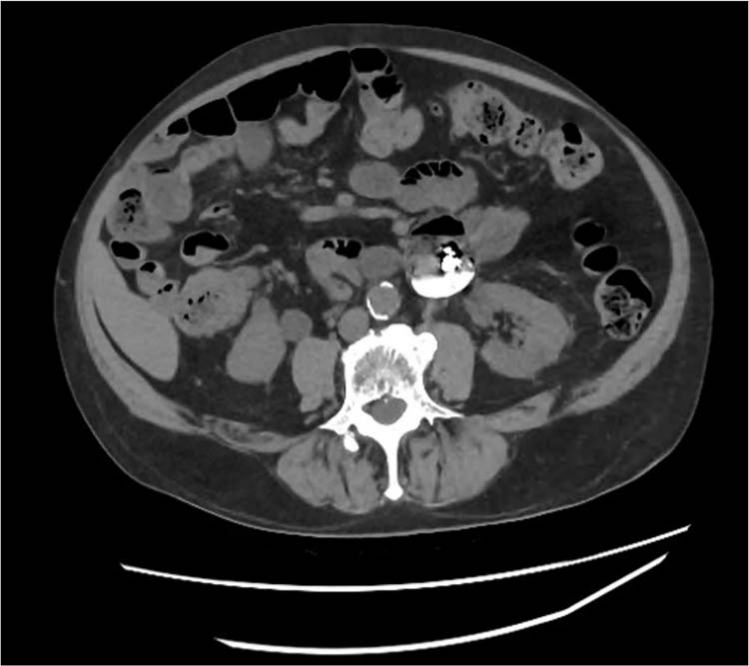
Abdominal CT identifying one hearing aid in a jejunal diverticulum 3 weeks after initial presentation – axial view.

**Figure 3:**
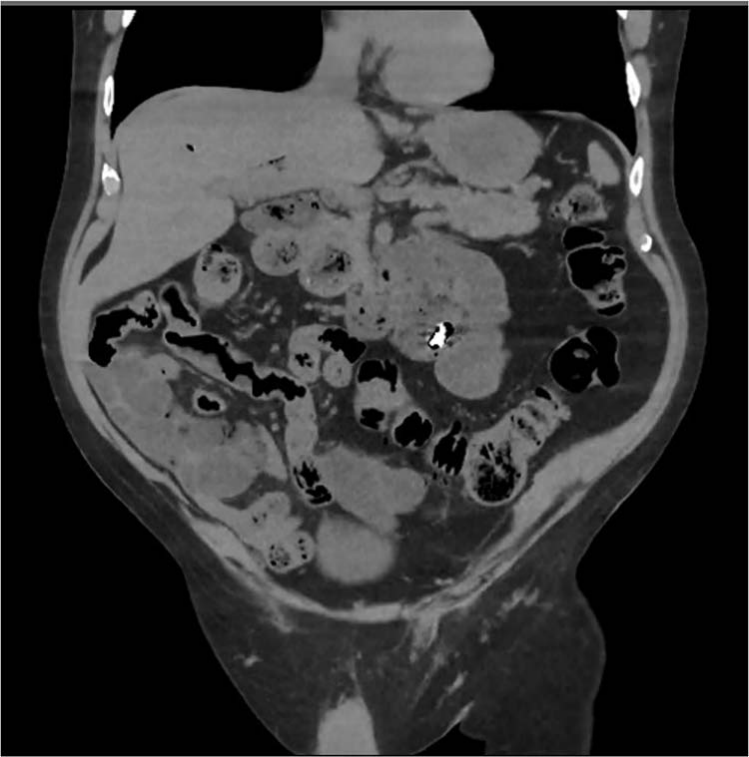
Abdominal CT identifying one hearing aid in a jejunal diverticulum 3 weeks after initial presentation – coronal view.

An 80 year old male presented to the emergency department after accidentally swallowing his hearing aids. On presentation he was pain free, hemodynamically stable, and had a soft abdomen. He had a background of type-2 diabetes, rheumatoid arthritis and diverticulitis. The hearing aids were powered by lithium battery. X-ray found two foreign bodies in the left upper abdomen (Fig. 1). After assessment in the emergency department, he returned home to await their natural passage. One hearing aid was found in his stools the following day, but he represented three weeks later as the second hearing aid remained unaccounted for. He underwent repeat abdominal x-ray and CT, which found the hearing aid retained within a presumed distal duodenal diverticulum (Figs 2 and 3). He was booked a push enteroscopy the following day.

**Figure 4:**
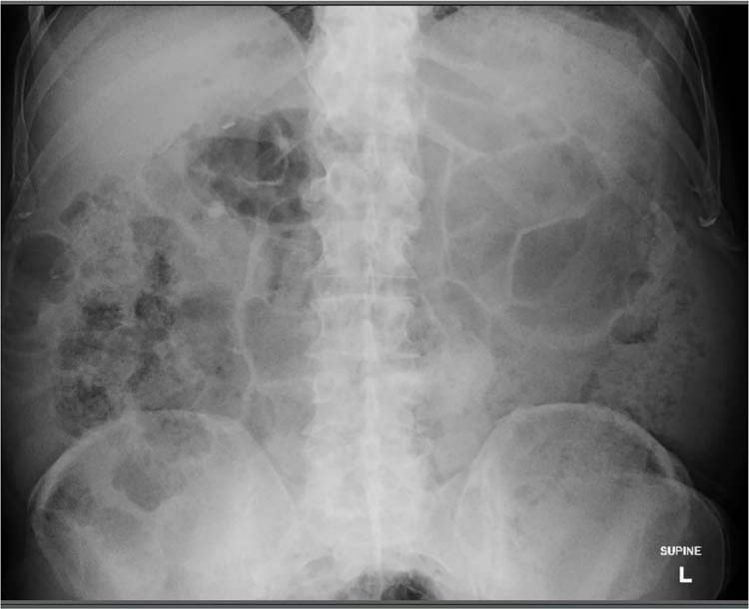
Abdominal X-ray demonstrating passage of second hearing aid.

**Figure 5:**
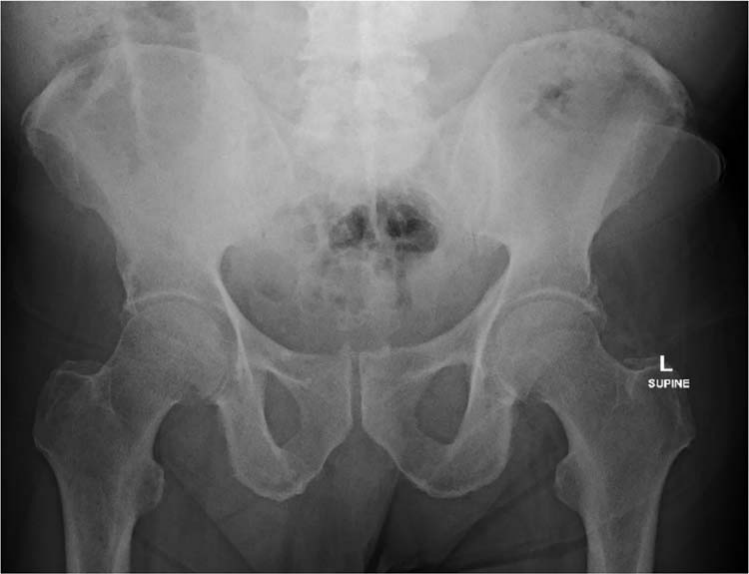
Pelvic X-ray demonstrating passage of second hearing aid.

During the push enteroscopy the hearing aid could not be found despite thorough inspection of duodenal diverticula. The procedure was assisted by image intensifier guidance, and upon consultation with the onsite radiologist, the impression was that the hearing aid was in a jejunal diverticulum rather than a duodenal diverticulum. The procedure was abandoned and a single balloon assisted enteroscopy was planned for the following day. Prior to the procedure, the foreign body could not be located on the initial scout x-ray, and it was presumed to have passed naturally. Follow up formal x-rays again did not identify a foreign body (Figs 4 and 5), and the patient has been well since.

## DISCUSSION

It is likely that formation of jejunal diverticula is due to a similar process to that of sigmoid diverticula – mucosal outpouching at the entry point of a blood vessel [1]. Intestinal dysmotility and high intraluminal pressures are likely involved [2], and familial aggregation has been observed [3, 4].

Foreign bodies that cause perforation of the gut are generally food products of animal or vegetable origin. Metallic or sharp objects often pass without intervention. Foreign body perforation of jejunal diverticula is rare in comparison to Meckel’s diverticula, despite a similar incidence of diverticula at the two sites [1]. This may be due to the comparatively wider neck of jejunal diverticula, allowing inflow and outflow, with less likelihood of foreign bodies getting stuck, and subsequent necrosis and perforation.

Five cases of foreign body perforation of jejunal diverticula have been described in the literature. One was due to a wooden stick, and caused rectal bleeding [5]. All others were due to food products, including fish bones [1, 6] a steak bone [7], and a vegetable stalk [8]. There have been reports of button batteries causing perforation of Meckel’s diverticula, with one due to an alkaline hearing aid battery [9]. There have been no documented cases of hearing aids or lithium batteries getting stuck in small bowel diverticula, therefore this was a novel situation. On review of the literature, retained food products in jejunal diverticula are likely to cause further complications, especially in symptomatic patients who have presented late. This case suggests that small, blunt retained foreign bodies retained in jejunal diverticula may pass naturally over the course of a few weeks, but require close observation even in the asymptomatic patient.
